# A Reversible Rocksalt to Amorphous Phase Transition Involving Anion Redox

**DOI:** 10.1038/s41598-018-33518-4

**Published:** 2018-10-10

**Authors:** Atsushi Sakuda, Koji Ohara, Tomoya Kawaguchi, Katsutoshi Fukuda, Koji Nakanishi, Hajime Arai, Yoshiharu Uchimoto, Toshiaki Ohta, Eiichiro Matsubara, Zempachi Ogumi, Kentaro Kuratani, Hironori Kobayashi, Masahiro Shikano, Tomonari Takeuchi, Hikari Sakaebe

**Affiliations:** 1 Research Institute of Electrochemical Energy, Department of Energy and Environment, National Institute of Advanced Industrial Science and Technology (AIST), 1-8-31 Midorigaoka, Ikeda, Osaka 563-8577 Japan; 2 Office of Society-Academia Collaboration for Innovation, Kyoto University, Gokasho, Uji, Kyoto 611-0011 Japan; 3 The Research & Utilization Division, Japan Synchrotron Radiation Research Institute (JASRI), 1-1-1 Kouto, Sayo, Hyogo 679-5198 Japan; 4 Graduate School of Human and Environmental Studies, Kyoto University, Nihonmatsu-cho, Yoshida, Sakyo-ku, Kyoto 606-8317 Japan; 5 SR Center, Ritsumeikan University, 1-1-1 Noji-Higashi, Kusatsu, Shiga 525-8577 Japan; 6 Present Address: Department of Applied Chemistry, Graduate School of Engineering, Osaka Prefecture University, 1-1 Gakuen-cho, Naka-ku, Sakai, Osaka 599-8531 Japan; 7 Present Address: Institute for Materials Research, Tohoku University, 2-1-1 Katahira, Aoba-ku, Sendai, Miyagi 980-8577 Japan; 8 Present Address: Department of Chemical Science and Engineering, School of Materials and Chemical Technology, Tokyo Institute of Technology, 4259 Nagatsuta, Midori-ku, Yokohma 226-8502 Japan; 9 Present Address: SR Center, Ritsumeikan University, 1-1-1 Noji-Higashi, Kusatsu, Shiga 525-8577 Japan

## Abstract

The charge-discharge capacity of lithium secondary batteries is dependent on how many lithium ions can be reversibly extracted from (charge) and inserted into (discharge) the electrode active materials. In contrast, large structural changes during charging/discharging are unavoidable for electrode materials with large capacities, and thus there is great demand for developing materials with reversible structures. Herein, we demonstrate a reversible rocksalt to amorphous phase transition involving anion redox in a Li_2_TiS_3_ electrode active material with NaCl-type structure. We revealed that the lithium extraction during charging involves a change in site of the sulfur atom and the formation of S−S disulfide bonds, leading to a decrease in the crystallinity. Our results show great promise for the development of long-life lithium insertion/extraction materials, because the structural change clarified here is somewhat similar to that of optical phase-change materials used in DVD-RW discs, which exhibit excellent reversibility of the transition between crystalline and amorphous phase.

## Introduction

As next generation batteries with high energy densities, lithium/sulfur batteries have been widely studied^1^. Among these are lithium/metal-polysulfide batteries, which have high theoretical energy densities and utilize metal polysulfides instead of sulfur or lithium sulfide^2–9^. Conventional lithium/sulfur batteries suffer from a few drawbacks, such as a low volumetric energy density, because they require a large amount of carbon to add electronic conductivity. The choice of electrolytes for lithium/sulfur batteries is limited, owing to adverse side-reactions. In contrast to sulfur electrodes, metal polysulfides have several advantages, such as a high energy density, because of their higher specific gravity and electronic conductivity. Furthermore, a large range of potential electrolytes can also be used in lithium/metal polysulfide batteries, insofar as metal polysulfides show low solubility in many organic solvents, and because undesirable side-reactions are suppressed^7^.

Mechanochemically-prepared Li_2_TiS_3_ and Li_3_NbS_4_ have a cation disordered cubic rocksalt structure and can charge/discharge with a capacity greater than 400 mAh g^−1^ ^5^. Upon 3.0 V (vs. Li^+^/Li) charging and 1.5 V discharging, approximately 2.6 and 3.5 lithium ions can reversibly insert into and extract from the structure with a composition range of 0.4 < *x* < 3.0 for Li_*x*_TiS_3_ and 0.4 < *y* < 4.0 for Li_*y*_NbS_4_, respectively. These materials show excellent cycle performance in all-solid-state lithium cells^5,6^. Amorphization occurs during the extraction of lithium from Li_2_TiS_3_, whereas reverse crystallization occurs during the lithium insertion process. Interestingly, the cation disordered cubic rocksalt-type phase, which should be metastable at room temperature, is re-formed after lithium insertion. Moreover, the phase transition should accompany anion redox. The nature of the rocksalt/amorphous phase transition has not yet been clarified. Elucidating the mechanisms of the charge-discharge process and structural change offers promise for designing long-life electrode materials with high energy densities because the elucidation of a novel charge-discharge mechanism and its applications have been a major breakthrough in their design, leading to new ideas for boosting their performance^7,10–16^. We previously reported the reversible anion redox in the amorphous titanium polysulfide electrode by X-ray pair distribution function (PDF) analyses and ab initio molecular dynamics (AIMD) calculations^7^. X-ray PDF analysis is a powerful technique for investigating the structure of amorphous materials. Thus, it is useful to elucidate the structural changes involving amorphization.

Here, we reveal a mechanism of the transition between the rocksalt and amorphous phases in Li_2_TiS_3_, including noticeable anion redox by PDF analyses and AIMD calculations.

## Results and Discussion

**Figure 1:**
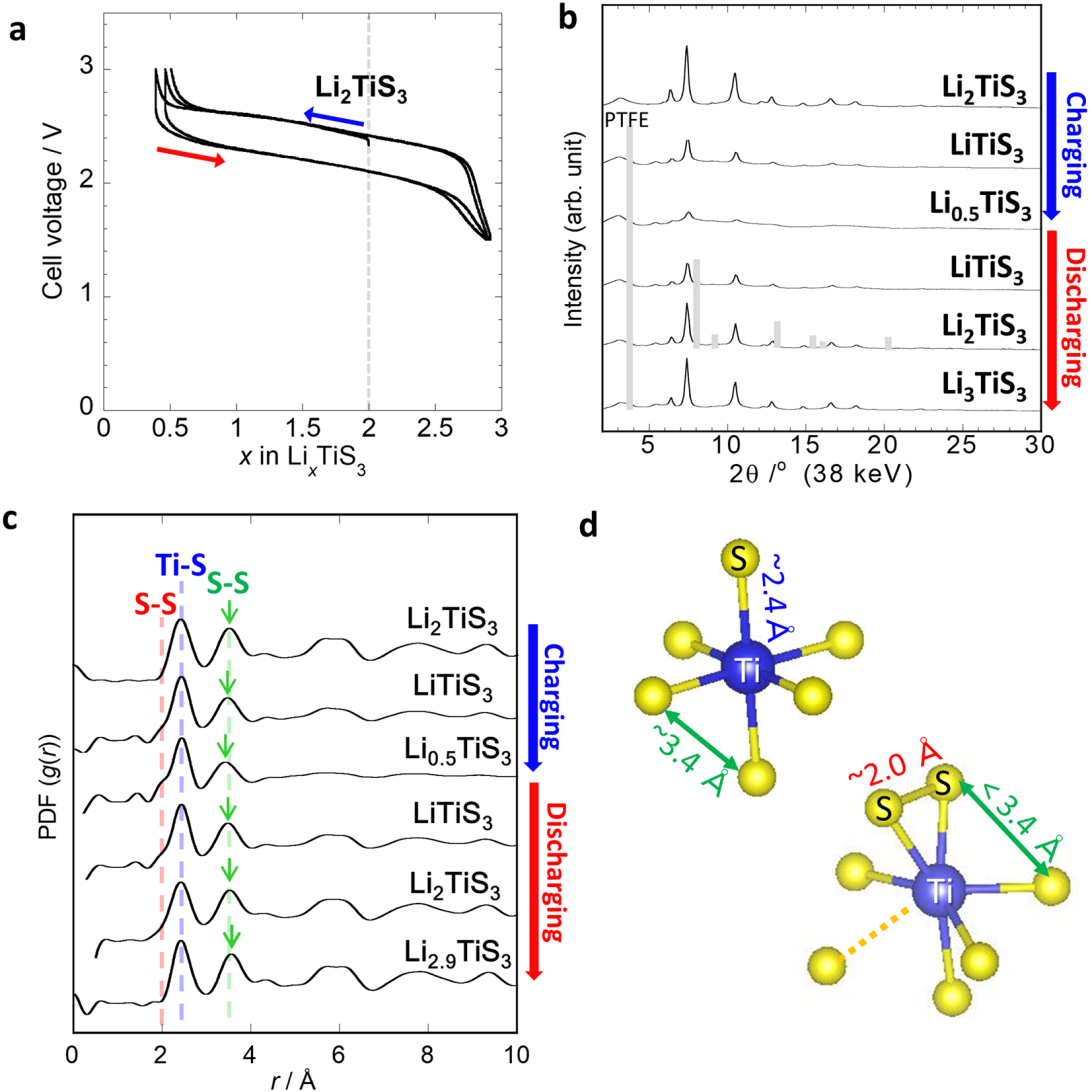
(**a**) Charge-discharge curves of cubic Li_2_TiS_3_, (**b**) XRD patterns after charging and discharging of cubic Li_2_TiS_3_, (**c**) PDF profiles after charging and discharging of cubic Li_2_TiS_3_, and (**d**) local structure model of Li_2_TiS_3_ before (upper) and after (bottom) charging.

Figure 1 shows the charge–discharge curves, the X-ray diffraction (XRD) patterns, and the PDF profiles following charging and discharging of cubic Li_2_TiS_3_. The pattern shows that Li and Ti randomly share the cation site, as we reported previously^5^. The peak intensities for cubic Li_2_TiS_3_ decreased when charging and increased when discharging, and a shift in the peak positions was barely observed, showing that the Li_2_TiS_3_ became amorphous upon charging, which agrees with our previous report^5^. The PDF analysis showed similar results; the middle-range order (>5 Å) decreased during charging and increased during discharging. The analysis of the correlations of atoms at short range (<5 Å) reveals the local structural changes involved in the amorphization. Based on an AIMD simulation, we previously reported that the S−S distance in homopolar S−S bonds was 2.0 Å, the Ti−S distance was around 2.4 Å, and the S−S distance of S coordinating to the same Ti was around 3.4 Å, as illustrated in Fig. 1d ^7^. These assignments can also be estimated from the crystal structure and Shannon’s radii and basically agreed with the results of AIMD simulations in this study. The peak (shoulder) of the S−S bonds appeared upon charging and disappeared upon discharging, indicating that the covalent S−S bonds reversibly formed and dissociated during the charge-discharge process. These analyses clearly showed two important results: (i) the charge-discharge mechanism involves a phase transition between the disordered rocksalt and amorphous phases, with the short- to middle-range structural change being essentially reversible in a wide composition range from Li_0.5_TiS_3_ to Li_3_TiS_3_, and (ii) the S−S bonds are formed and dissociated during charging and discharging, respectively, showing that the charge-discharge mechanism involves the redox of sulfur. The PDF analyses showed another unusual result: the Ti−S distance remains essentially unchanged during charging and discharging; however, the S−S distance at around 3.4 Å slightly shortens and lengthens during charging and discharging, respectively. This suggests that the coordination environments of both Ti for S and S for Ti change only slightly during charging and discharging. That is, the coordination number of Ti for S was kept at nearly 6 after charging. This is in contrast to the behavior of amorphous TiS_4_ electrodes. Indeed, we previously reported that the coordination number of S around Ti continually changes in an amorphous TiS_4_ electrode^7^. The X-ray absorption near edge structure (XANES) spectra of S K-edge of Li_2_TiS_3_ and the charge-discharge products were shown in Fig. S1. The reversible changes in the peak intensity and a shift of the absorption edge energy were observed in the spectra, indicating that the electron state of sulfur reversibly changed during charging and discharging.

**Figure 2:**
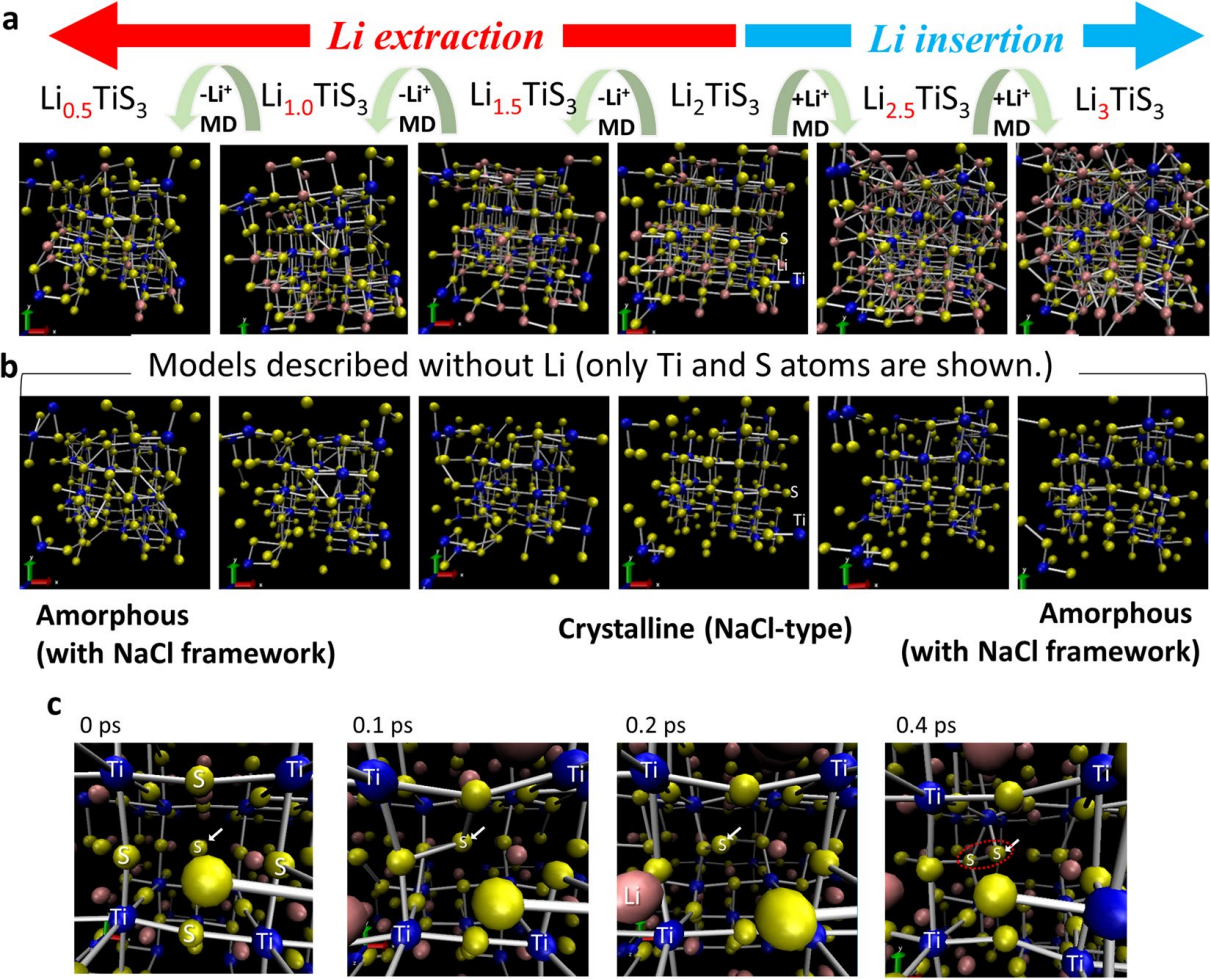
(**a**) Structural model of Li_*x*_TiS_3_ prepared by lithium extraction or insertion using AIMD simulations; (**b**) models described without Li (only Ti and S atoms are shown); and (**c**) snapshots of the shifted sulfur during MD simulations.

**Figure 3:**
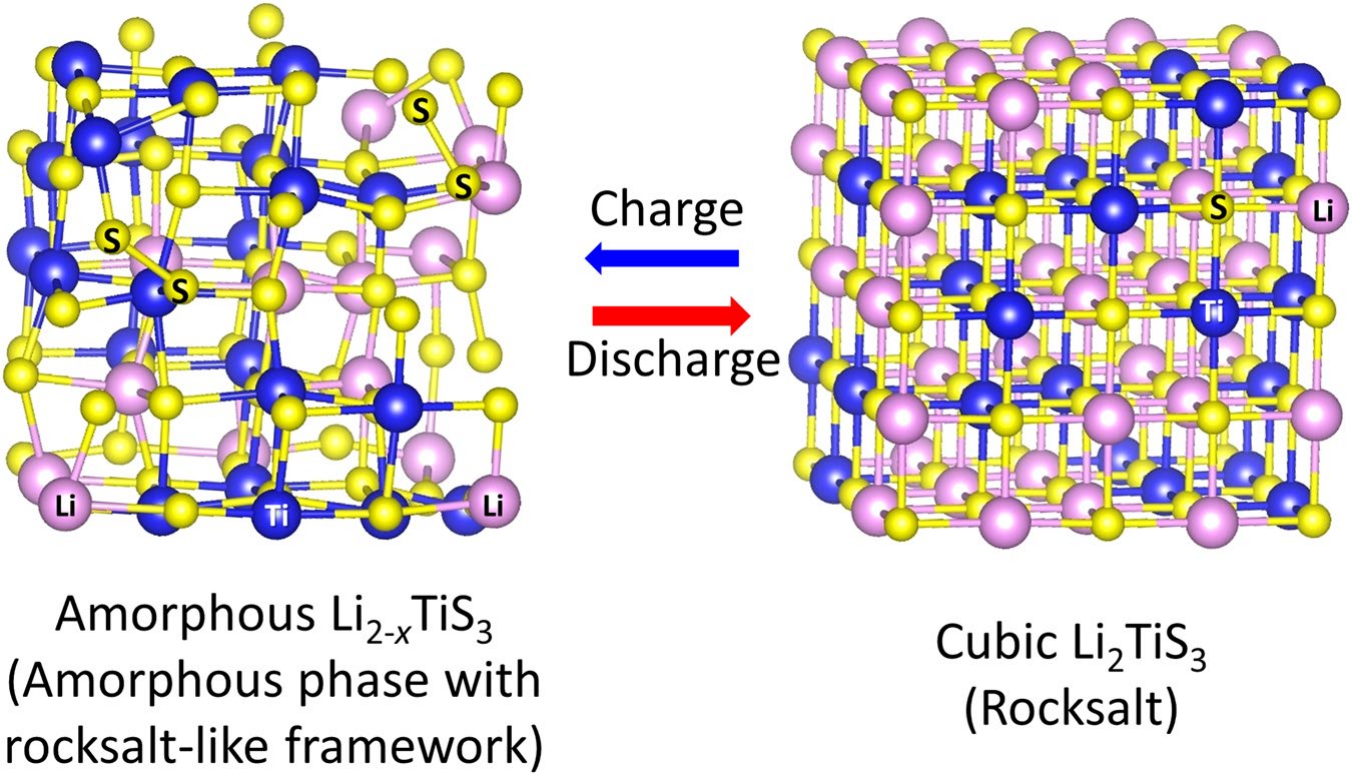
Schematic of structural change during charging and discharging.

We employed computational methods to visualize structural changes during charging/discharging, and these methods are effective at showing the changes that occur in low-crystalline materials^7^. In this study, the AIMD simulations offered a suitable model to explain the experimental results. Figure 2 shows the AIMD simulations of the structural model of Li_*x*_TiS_3_ prepared by lithium extraction/insertion. We constructed a 3 × 3 × 3 super cell of cubic rocksalt Li_2_TiS_3_ with 216 atoms and extracted randomly selected Li atoms to evaluate the structure of Li_2-*x*_TiS_3_. After cell parameter optimization, the structure was relaxed by AIMD simulations to obtain the structural models. The lithium excess models were calculated similarly. Figure 2a,b show that the atom ordering in the rocksalt structure decreased with lithium extraction. That is, crystallinity decreased, with the structure becoming amorphous. Conversely, the positions of Ti, Li, and other S atoms were nearly unchanged and the nature of the bonding between them was also almost unchanged. Some of the S atoms moved and formed S−S bonds upon lithium extraction, as shown in Fig. 2c. These results clearly explained why only a slight shift in the XRD peaks was observed and the Ti−S distance was almost unchanged in the PDF profiles; the framework (rocksalt-like structure) formed by the Ti−S−Ti network did not change dramatically. The randomness of Li/Ti and the maintained framework also contributed to the amorphization. The disorder of Li/Ti induced a variation in the Li potential, resulting in random extraction of Li with a lower electrochemical potential from the rocksalt structure during charging. As Li atoms were randomly extracted, the rearrangement of the structure was suppressed, and thus its amorphous structure was maintained. This is why the phase transition occurred between the crystalline and amorphous phases rather than the crystalline and crystalline phases. These results indicated that the lithium-extracted Li_*x*_TiS_3_ was the material between the crystal and amorphous phases, and that the crystallinity changed gradually and continuously with the Li composition. The schematics of the structural models of amorphous Li_2-*x*_TiS_3_ and cubic Li_2_TiS_3_ are shown in Fig. 3. Electrode materials with a large capacity unavoidably undergo large and usually irreversible structural changes during lithium insertion/extraction, due to the large change in volume. In the phase transition shown here, the volume change is somewhat suppressed as a result of increasing the free volume.

As expected, S atoms that lost more than one neighbour Li preferentially changed their positions to form homopolar S−S bonds, which means that S is oxidized during charging. Furthermore, most disulfides (S−S) directly interact with Ti or Li. Before and after relaxation, the partial density of states (PDOS) of S atoms which form S−S bonds dramatically changed (see Fig. S2), indicating that the charge was compensated for by the S atoms that formed S−S bonds (see Figs S2 and S3, and Table S1). Furthermore, the change in partial atomic charge (Mulliken charge) also showed that the loss of charge due to lithium extraction was compensated for by all the atoms surrounding the extracted lithium. In the cation disordered lithium transition metal sulfides with a lithium-rich composition, the tetrahedron site becomes a diffusion pathway when next neighbour of the site is surrounded by Li, and when the volume of the tetrahedron site is large, as reported by Lee *et al*.^17^. Amorphization increases the free volume and distorts the tetrahedral site, facilitating the extraction of Li. This is the reason why a large amount of Li can be extracted from the cubic rocksalt structure.

Unlike in the case of many oxide materials, disulfides formed in the sulfide are accommodated in the framework, and thus showed high reversibility even in deep charging. The results obtained by computational studies were consistent with the experimental results of XRD and PDF analyses. The mechanism elucidated here will be applicable to the study of oxygen^18^ and other anion redox couples, depending on the conditions.

With the Li-rich composition of Li_2+*x*_TiS_3_, the number of cations becomes larger than that of anions. In this case, it is assumed that a rocksalt structure can no longer be maintained, and that a large structural phase transition will occur. Nevertheless, the rocksalt phase was detected in this composition. It is thought that the peaks in the XRD pattern in Fig. 1b can largely be attributed to Li_2_TiS_3_ and amorphous Li_2+*x*_TiS_3_ formed in the discharged electrodes, rather than crystalline Li_2+*x*_TiS_3_. The AIMD simulation suggests a decrease in crystallinity, as shown in Fig. 2. Further studies are needed to determine whether the phase containing additional lithium is amorphous or whether sulfur defects are involved.

Interestingly, the transition between the rocksalt and amorphous phases examined in this study partially resembles that in optical phase transition materials, which are used in DVD-RW and DVD-RAM discs^19–21^. In a representative optical phase transition material—viz., Ge_2_Sb_2_Te_5_—the Ge-Te-Ge network is a core network in both crystalline (disordered rocksalt) and amorphous phases, which contributes to rapid and highly reversible phase transition. The Sb-Te bond is relatively weak and fragile, and acts as a trigger for crystallization and amorphization^21^. The role of the Ti-S-Ti network in this study is similar to that of the Ge-Te-Ge core network in the phase transition material. The behaviour of the Li-S and S-S bonds is also similar to that of the Sb-Te bond. In optical phase change materials, rapid and excellent reversibility has been achieved over 10^6^ cycles. Thus, it is expected that high-power electrode materials with an ultra-long cycle life can be developed based on the idea of mimicking optical phase-change materials.

## Conclusion

In this paper, we described the mechanism of the reversible rocksalt to amorphous phase transition involving anion redox in cubic Li_2_TiS_3_. Extracting lithium while charging involves the oxidation of sulfur and the formation of S−S disulfide bonds; the formation of a disulphide bond leads to a decrease in crystallinity. The reverse structural change occurs when discharging. Although cubic Li_2_TiS_3_ is a metastable phase, the phase is preferably formed during lithium insertion after amorphization by lithium extraction, because the amorphous phase contains a cubic rocksalt analogue Ti-S-Ti core framework. The core framework forms an intermediate state between amorphous and crystal. We also found similarities between the phase transition in the electrode material of this study and that of optical phase transition materials. Anion redox combined with the mimicry of optical phase-change materials is thus a promising approach to the future development of novel large-capacity and ultra-long-life electrode materials.

## Methods

### Materials

Cubic Li_2_TiS_3_ was prepared by a mechanochemical process^5^. Crystalline TiS_2_ (99.8%, Aldrich) and Li_2_S (99.9%, Mitsuwa Pure Chemicals) were used as the starting materials. Zirconia pots (45 mL) and 500 zirconia balls (4 mm in diameter) were used for ball-milling; the planetary ball-mill apparatus (P-7, Fritsch) was operated at 510 rpm for 40 h.

### Electrochemical cells

To prepare the Li_2_TiS_3_ working electrode, Li_2_TiS_3_, acetylene black (AB) and polytetrafluoroethylene (PTFE) were mixed in an agate mortar^5^. The weight ratio of Li_2_TiS_3_, AB and PTFE was 9:1:1. The working electrode was attached to an Al mesh current corrector. The loading rate of the active material was ~20 mg cm^−2^. A 1 M solution of LiPF_6_ in a 50:50 (by volume) mixture of ethylene carbonate and dimethyl carbonate (Tomiyama Pure Chemical Industries Ltd.) was used as the electrolyte, and a piece of Li foil was used as the counter electrode. The electrochemical measurements were performed at 30 °C using a charge-discharge unit (TOSCAT-3100, Toyo System) at a current density of 20 mA g^−1^.

### High-energy X-ray diffraction measurements

The X-ray total scattering measurements for the PDF analysis were carried out at SPring-8 BL28XU^22^. The incident X-ray energy was 38.0 keV, and a Si (220) crystal (2*d* = 3.840 Å) monochromator was used. The analysed Q-range was 0.7–14 Å^−1^. The intensity of the incident X-rays was monitored in an ionisation chamber filled with Ar gas, while the scattered X-rays were detected with a YAP detector. A vacuum chamber was used to suppress air scattering. The collected datasets were corrected for the absorption, background and polarisation effects. Details of the correction and normalisation procedures have been reported elsewhere^23^.

### DFT-MD simulations

The AIMD were performed using OpenMX (ver. 3.8)^24^. The exchange-correlation functional used was GGA-PBE. The cut-off energy was 300 Ry. The basis functions (cut-off radius/orbital structure) used were 7.0 Bohr/s^3^p^3^d^2^ (Ti), 7.0 Bohr/s^2^p^2^d^1^ (S) and 8.0 Bohr/s^3^p^2^ (Li). A norm-conserving pseudopotential was used. For the MD calculations, the O(N) Krylov-subspace method was used^25^. The MD temperature was controlled to 400 K, and the time step was 1.2 fs/MD-step. The FOCUS supercomputer system was used for the calculations. The atomic models were described using VESTA software^26^.

3 × 3 × 3 super cells of cubic rock-salt Li_2_TiS_3_ (216 atoms) were constructed by total energy calculation. The lowest energy configuration in the calculated model was determined from 20 different configurations. Randomly selected Li atoms were then extracted and the cell parameter with the lowest energy was recalculated. Finally, the structure was relaxed by AIMD at 400 K and optimized at 0 K. The lithium extraction and relaxation process was repeated several times.

### X-ray absorption fine structure (XAFS) measurements

The sulfur K-edge XAFS measurements were carried out at BL-10 of the SR Center, Ritsumeikan University. The partial fluorescence yield (PFY) mode was used to obtain the spectra with a silicon drift detector. The incident X-ray beam was monochromatised with a Ge(111) crystal (2*d* = 6.532 Å). The absolute photon energy was calibrated with the assumption that the strong resonance of K_2_SO_4_ (S 1*s* → *t*2) appears at 2481.7 eV.

## Electronic supplementary material

Supplementary information
