# Supplementary material for: A Reversible Rocksalt to Amorphous Phase Transition Involving Anion Redox
Source: Sci Rep. 2018 Oct 10;8:15086. doi: 10.1038/s41598-018-33518-4 (PMC6180042; doi:10.1038/s41598-018-33518-4)
Supplement: Supplementary file 1 — Supplementary information [file 41598_2018_33518_MOESM1_ESM.pdf]

A Reversible Rocksalt to Amorphous Phase Transition Involving Anion Redox

Atsushi Sakuda[1,*,+], Koji Ohara[2,3], Tomoya Kawaguchi[2], Katsutoshi Fukuda[2], Koji Nakanishi[2], Hajime Arai[2], Yoshiharu Uchimoto[4], Toshiaki Ohta[5], Eiichiro Matsubara[2], Zempachi Ogumi[2], Kentaro Kuratani[1], Hironori Kobayashi[1], Masahiro Shikano[1], Tomonari Takeuchi[1,*] and Hikari Sakaebe[1]

[1] Research Institute of Electrochemical Energy, Department of Energy and Environment, National Institute of Advanced Industrial Science and Technology (AIST), 1-8-31 Midorigaoka, Ikeda, Osaka, 563-8577, Japan

[2] Office of Society-Academia Collaboration for Innovation, Kyoto University, Gokasho, Uji, Kyoto, 611-0011, Japan

[3] The Research & Utilization Division, Japan Synchrotron Radiation Research Institute (JASRI), 1-1-1 Kouto, Sayo, Hyogo, 679-5198, Japan

[4] Graduate School of Human and Environmental Studies, Kyoto University, Nihonmatsu-cho, Yoshida, Sakyo-ku, Kyoto, 606-8317, Japan

[5] SR Center, Ritsumeikan University, 1-1-1 Noji-Higashi, Kusatsu, Shiga, 525-8577, Japan

[+] Present address: Department of Applied Chemistry, Graduate School of Engineering, Osaka Prefecture University, 1-1 Gakuen-cho, Naka-ku, Sakai, Osaka, 599-8531, Japan

Corresponding Author*

A.S.: saku@chem.osakafu-u.ac.jp

T.T.: takeuchi.tomonari@aist.go.jp

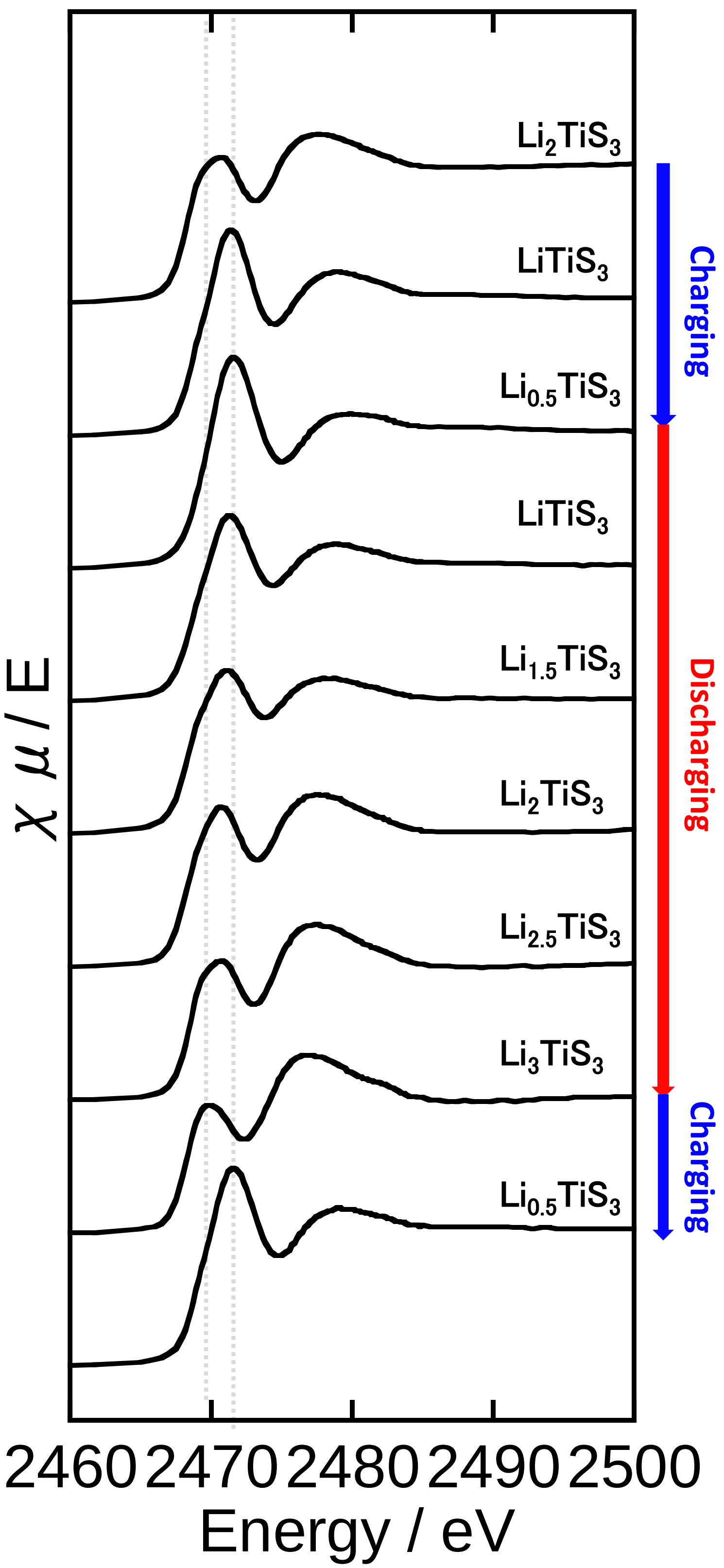

Figure S1 XANES spectra of S K-edge of Li_2TiS_3 and the charge-discharge products.

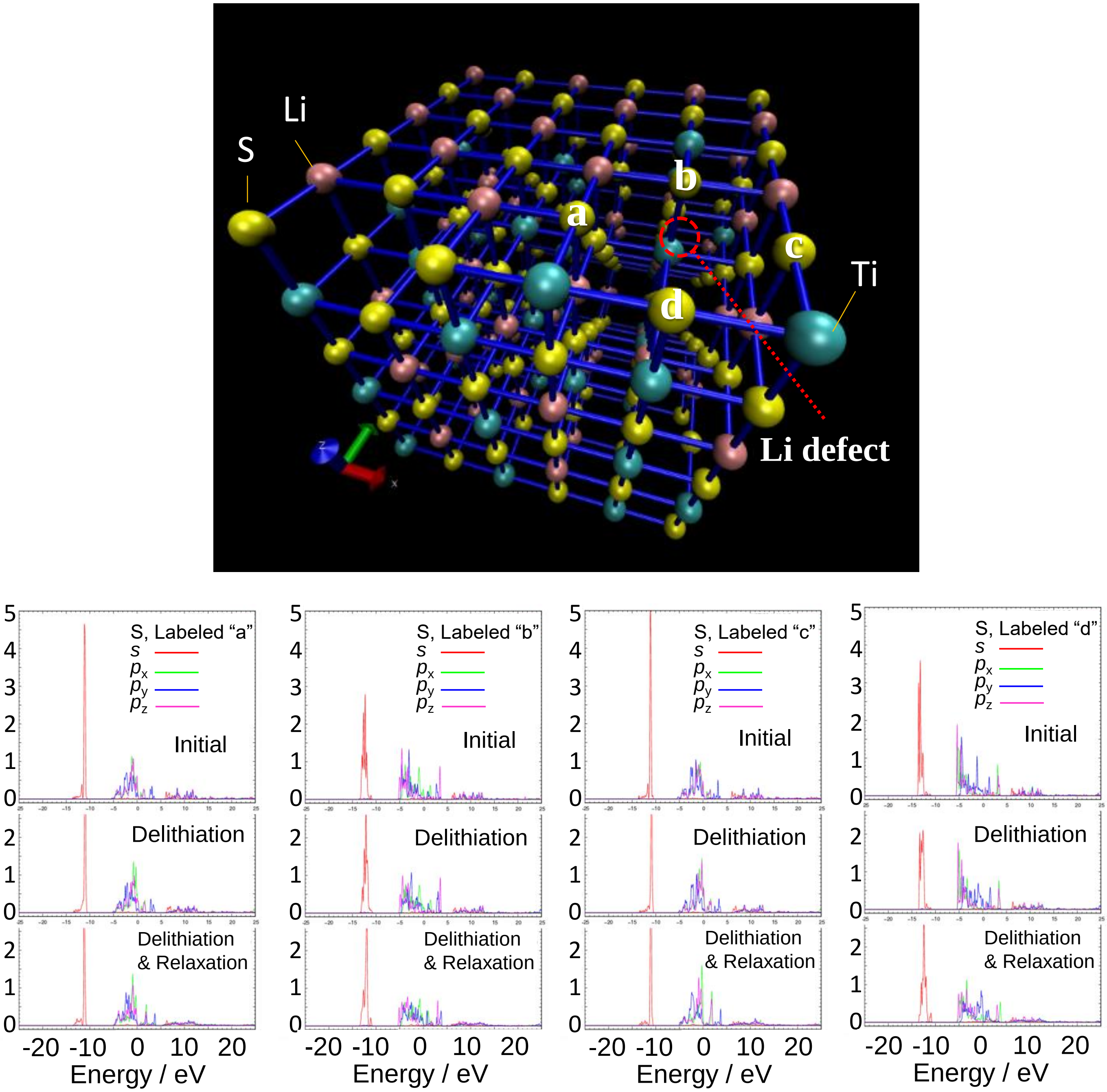

Figure S2 Change of partial density of states (PDOS) of “unmoved” sulfur atoms by relaxations.
a: S with coordination of 4 Li / 1 Ti / 1 vacancy.
b: S with coordination of 2 Li / 3 Ti / 1 vacancy.
c: S with coordination of 3 Li / 1 Ti / 2 vacancy.
d: S with coordination of 1 Li / 4 Ti / 1 vacancy.
Upper: PDOS of initial state labeled sulfurs without lithium defects
Middle: PDOS of labeled sulfurs after delithiation without relaxations.
Bottom: PDOS of labeled sulfurs after delithiation and relaxations.

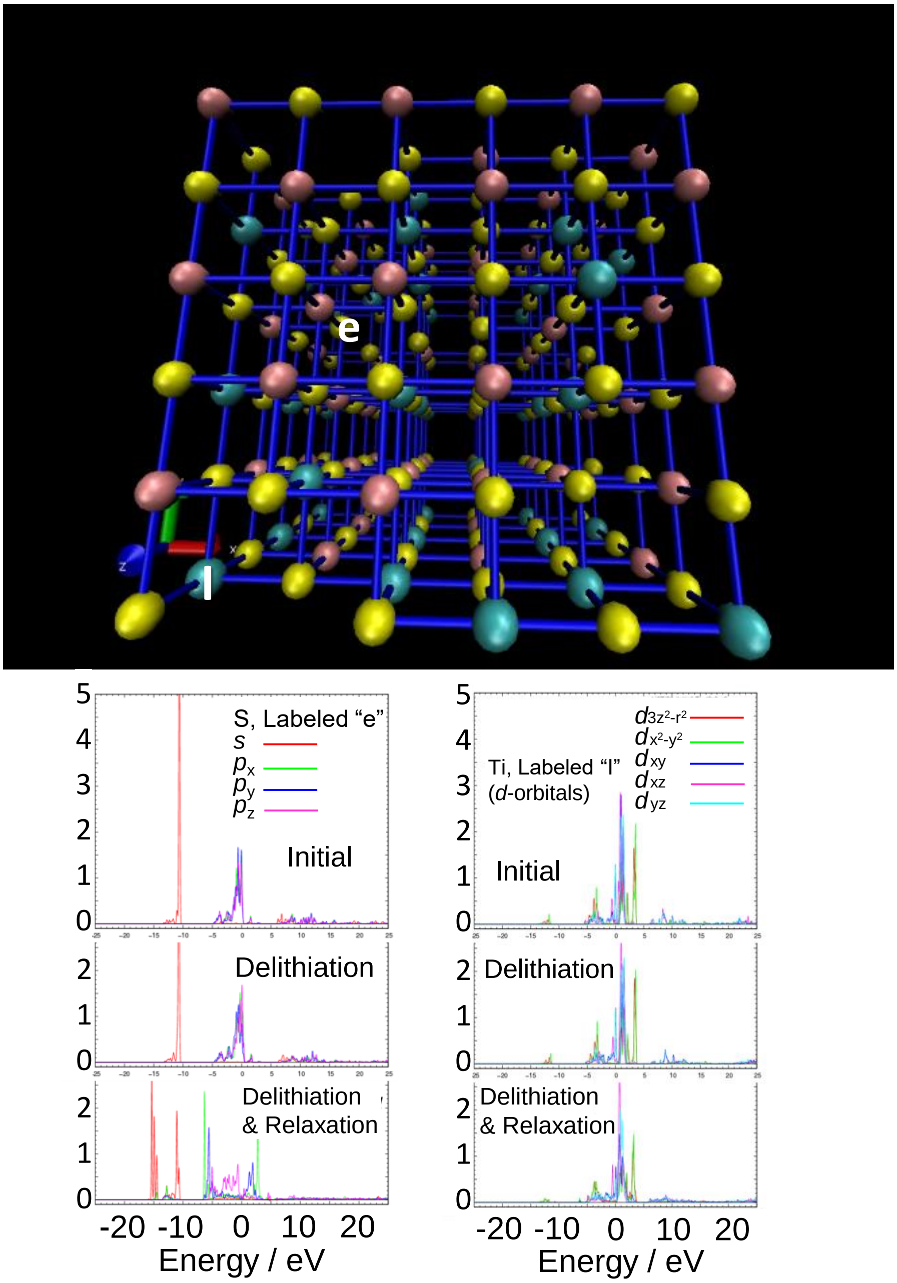

Figure S3 Change of partial density of states (PDOS) of "moved" sulfur and titanium atoms by relaxations.
e: S with coordination of 4 Li / 0 Ti / 2 vacancy.
I: Ti with coordination of 6 S.
Upper: PDOS of initial state labeled atoms without lithium defects
Middle: PDOS of labeled atoms after delithiation without relaxations.
Bottom: PDOS of labeled atoms after delithiation and relaxations.

Table S1 Calcurated Mulliken charges of S(a-e) and Ti(I).

Black: initial, purple: after delithiation, and red: after delithiation and relaxations.

	s			*p*			*d*		Σ	*Ionic Valence*
	0	1	Σ	0	1	Σ	0	Σ		
**S (a)**	**1.8495**	**0.0052**	**1.8546**	**4.3853**	**0.0406**	**4.4259**	**0.0714**	**0.0714**	**6.3519**	**-0.3519**
	**1.8672**	**0.0008**	**1.8681**	**4.3373**	**0.0179**	**4.3552**	**0.0698**	**0.0698**	**6.2931**	**-0.2931**
	**1.8505**	**0.0020**	**1.8524**	**4.3606**	**0.0267**	**4.3873**	**0.0806**	**0.0806**	**6.3203**	**-0.3203**
**S (b)**	**1.8168**	**0.0211**	**1.8379**	**4.3612**	**0.0650**	**4.4263**	**0.1202**	**0.1202**	**6.3844**	**-0.3844**
	**1.8294**	**0.0189**	**1.8483**	**4.3249**	**0.0543**	**4.3793**	**0.1143**	**0.1143**	**6.3419**	**-0.3419**
	**1.8349**	**0.0200**	**1.8549**	**4.3087**	**0.0560**	**4.3647**	**0.1083**	**0.1083**	**6.3279**	**-0.3279**
**S (c)**	**1.8491**	**0.0050**	**1.8541**	**4.3866**	**0.0400**	**4.4265**	**0.0713**	**0.0713**	**6.3519**	**-0.3519**
	**1.8835**	**-0.0062**	**1.8773**	**4.2913**	**-0.0044**	**4.2869**	**0.0672**	**0.0672**	**6.2314**	**-6.2314**
	**1.8692**	**-0.0067**	**1.8625**	**4.3060**	**0.0106**	**4.3166**	**0.0795**	**0.0795**	**6.2587**	**-0.2587**
**S (d)**	**1.7956**	**0.0213**	**1.8169**	**4.3680**	**0.0680**	**4.4360**	**0.1571**	**0.1571**	**6.4099**	**-0.4099**
	**1.8028**	**0.0179**	**1.8206**	**4.3475**	**0.0530**	**4.4005**	**0.1467**	**0.1467**	**6.3678**	**-0.3678**
	**1.8187**	**0.0297**	**1.8484**	**4.2969**	**0.0656**	**4.3625**	**0.1191**	**0.1191**	**6.3300**	**-0.3300**
**S (e)**	**1.8769**	**0.0009**	**1.8778**	**4.3778**	**0.0191**	**4.3969**	**0.0562**	**0.0562**	**6.3310**	**-0.3310**
	**1.9182**	**-0.0065**	**1.9117**	**4.2460**	**-0.0142**	**4.2319**	**0.0559**	**0.0559**	**6.1994**	**-0.1994**
	**1.9153**	**0.0088**	**1.9241**	**3.8359**	**0.0058**	**3.8417**	**0.3079**	**0.3079**	**6.0738**	**-0.0738**

	s				*p*			
	0	1	2	Σ	0	1	2	Σ
**Ti [I]**	**1.9996**	**0.4234**	**0.0121**	**2.4351**	**5.9448**	**0.7576**	**-0.0126**	**6.6897**
	**2.0016**	**0.3734**	**0.0124**	**2.3873**	**5.9456**	**0.7173**	**-0.0159**	**6.6470**
	**2.0028**	**0.3668**	**0.0133**	**2.3829**	**5.9480**	**0.7133**	**-0.0125**	**6.6489**

	d			Σ	*Ionic Valence*
	0	1	Σ		
**Ti [I]**	**2.7536**	**-0.0275**	**2.7262**	**11.8510**	**0.1490**
	**2.7385**	**-0.0472**	**2.6913**	**11.7256**	**0.2744**
	**2.7333**	**-0.0465**	**2.6867**	**11.7185**	**0.2815**
